# Single-digit Arabic numbers do not automatically activate magnitude representations in adults or in children: Evidence from the symbolic same–different task^☆^

**DOI:** 10.1016/j.actpsy.2013.08.006

**Published:** 2013-11

**Authors:** Becky Wong, Dénes Szücs

**Affiliations:** Centre for Neuroscience in Education, Department of Psychology, University of Cambridge, Downing Street, Cambridge CB2 3EB, United Kingdom

**Keywords:** Symbolic numbers, Numerical cognition, Automaticity, Magnitude representations, Same–different task

## Abstract

We investigated whether the mere presentation of single-digit Arabic numbers activates their magnitude representations using a visually-presented symbolic same–different task for 20 adults and 15 children. Participants saw two single-digit Arabic numbers on a screen and judged whether the numbers were the same or different. We examined whether reaction time in this task was primarily driven by (objective or subjective) perceptual similarity, or by the numerical difference between the two digits. We reasoned that, if Arabic numbers automatically activate magnitude representations, a *numerical* function would best predict reaction time; but if Arabic numbers do not automatically activate magnitude representations, a *perceptual* function would best predict reaction time. Linear regressions revealed that a perceptual function, specifically, subjective visual similarity, was the best and only significant predictor of reaction time in adults and in children. These data strongly suggest that, in this task, single-digit Arabic numbers do not necessarily automatically activate magnitude representations in adults or in children. As the first study to date to explicitly study the developmental importance of perceptual factors in the symbolic same–different task, we found no significant differences between adults and children in their reliance on perceptual information in this task. Based on our findings, we propose that visual properties may play a key role in symbolic number judgements.

## Highlights

• Single-digit Arabic numbers do not automatically activate magnitude representations.
• This is true for both adults and children.
• Subjective visual properties may play a key role in symbolic number judgments.

## 1 Introduction

Most cultures employ the use of symbolic numbers, such as Arabic digits, to convey numerical magnitudes. Several researchers assume that the mere presentation of symbolic numbers automatically activates corresponding mental representations of magnitude in humans (Girelli, Lucangeli, & Butterworth, 2000; Tzelgov & Ganor-Stern, 2005). This contention was largely based on the results of behavioural studies that tested only one numerical hypothesis (e.g. Dehaene & Akhavein, 1995; Duncan & McFarland, 1980; Ganor-Stern & Tzelgov, 2008). These studies assumed that their experimental effects could only be explained by the numerical properties of the stimuli. In contrast, Cohen (2009) contrasted the numerical hypothesis with an alternative perceptual hypothesis. His approach examined whether the experimental effects could be explained by non-numerical (i.e. perceptual) rather than numerical properties of the stimuli. Recent studies based on this new approach have found evidence for the argument that the mere presentation of symbolic numbers does not automatically activate their magnitude representations (e.g., Defever, Sasanguie, Vandewaetere, & Reynvoet, 2012; Garcia-Orza, Perea, Abu Mallouh, & Carreiras, 2012; Lyons, Beilock, & Ansari, 2012). In this study, we further investigated whether single-digit Arabic numbers automatically activate magnitude representations in adults or in children by substantially extending the original experimental task (Cohen, 2009), stimuli, participants, explanatory functions tested, and analyses.

### 1.1 Background

It is widely thought that humans possess imprecise internal representations of magnitudes (henceforth referred to as magnitude representations) that correspond to symbolic numbers in the external environment (Domahs et al., 2012; Lyons & Ansari, 2008; Verguts & Fias, 2004; Young & Opfer, 2011; Zhang & Wang, 2005). Certainly, it seems that symbolic numbers may activate such magnitude representations. The classic evidence for this argument comes from the comparison distance effect that appears in the numerical comparison task with symbolic numbers (e.g., Buckley & Gillman, 1974; Moyer & Landauer, 1967). But, do symbolic numbers *automatically* activate magnitude representations? A strong test of an automatic process is to examine whether it occurs even when it is not directly relevant to the demands of the task at hand (Bugden & Ansari, 2011; Ganor-Stern & Tzelgov, 2008; Rubinsten, Henik, Berger, & Sharhar-Shalev, 2002; Tzelgov, Meyer, & Henik, 1992; Zhang, Si, Zhu, & Xu, 2010).

Early studies seemed to suggest that symbolic numbers may indeed automatically activate magnitude representations. One strand of evidence came from the matching distance effect in symbolic same–different tasks (e.g. Dehaene & Akhavein, 1995; Duncan & McFarland, 1980; Ganor-Stern & Tzelgov, 2008; Van Opstal & Verguts, 2011; Verguts & van Opstal, 2005). However, the matching distance effect does not appear to be highly reliable, as other studies have not found this effect in the same–different task (Cohen, 2009; Defever et al., 2012; Goldfarb, Henik, Rubinsten, Bloch-David, & Gertner, 2011; Sasanguie, Defever, Van den Bussche, & Reynvoet, 2011). A second strand of evidence came from the size congruity effect in the physical size decision Stroop paradigm (e.g. Cohen Kadosh et al., 2007; Girelli et al., 2000; Mussolin & Noel, 2008; Szűcs & Soltesz, 2007, 2008; Szűcs, Soltesz, Jarmi, & Csepe, 2007; Tzelgov et al., 1992; Zhou et al., 2007). The current study only deals with the symbolic same–different task.

### 1.2 Perceptual vs. numerical factors in the same–different task

In 2009, Cohen proposed a more nuanced approach to tackle the question of whether symbolic numbers automatically activate their magnitude representations. Although a relationship between numerical distance and reaction time clearly exists, Cohen (2009) suggested that there may be a relationship between the physical form of symbolic numbers and reaction time. To the extent that visual similarity and magnitude representations are correlated, Cohen (2009) contended that the studies of the symbolic same–different task, which investigated only one (numerical) hypothesis, might have missed a potential perceptual confound. He thus argued that researchers investigating this question should test two hypotheses: a numerical hypothesis and a perceptual hypothesis.

**Fig. 1:**
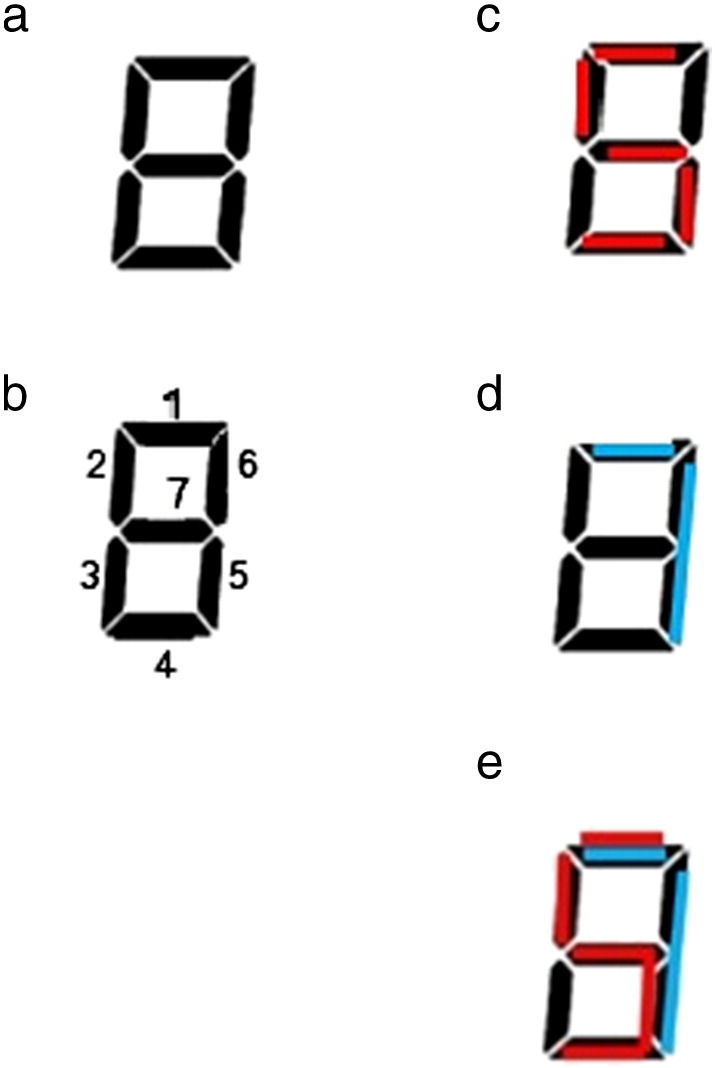
(a) The figure 8 structure as used in Cohen's (2009) perceptual function (P_C_). (b) This structure is made up of 7 lines. (c) The digit 5 is imposed on the structure. (d) The digit 7 as imposed on the structure. (e) A minimum of 6 lines are needed to form the digits 5 and 7. Two lines overlap. Four lines are used only once.

Cohen (2009) conducted a 5-or-not-5 task, in which adult participants were presented with single-digit Arabic numbers (henceforth referred to as probes) and made a button-press response as to whether each probe was 5 or not 5. As a measure of visual similarity between 5 and the probe, Cohen (2009) codified a perceptual function, abbreviated as P_C_ (Table 1). The formula was P_C_ = O/D, where O is the number of lines that the two digits share (i.e. overlapping lines), and D is the number of unshared lines between the two digits (i.e. difference) (Fig. 1). As a measure of magnitude processing, Cohen (2009) employed the well-known Welford (1960) function, abbreviated as N_W_ (Table 1). The formula for N_W_ was RT = a + k ∗ lg(L/L–S), where RT is the reaction time, L is the larger number, S is the smaller number, and a and k are constants.

Cohen (2009) then ran two separate linear regressions with reaction time as the criterion variable and either P_C_ or N_W_ as the predictor variable. To the extent that Arabic numbers automatically activate their corresponding magnitude representations, Cohen (2009) argued that the numerical function would dominate participants' responses even in a task which required no explicit magnitude judgements, i.e., N_W_ would be the best predictor of reaction time. Cohen (2009) further argued that Arabic numbers, minus the semantic (numerical) meaning they convey, are essentially symbolic shapes. Thus, to the extent that Arabic numbers do not automatically activate their corresponding magnitude representations, the function based on visual similarity, i.e., P_C_, would best predict reaction time. Both P_C_ and N_W_ separately and significantly predicted reaction time; however, P_C_ was a better fit for the data and accounted for a substantial percentage of the variance (93%). Thus, Cohen (2009) concluded that Arabic numbers do not automatically activate magnitude representations.

Garcia-Orza et al. (2012) applied Cohen's (2009) logic to investigate whether Persian and Arabic versions of Indian numbers automatically activate magnitude representations. The researchers recruited three groups of participants — Pakistani students, who were fluent only in the Persian version of Indian numbers; Jordanian students, who were fluent only in the Arabic version of Indian numbers; and Spanish students, who had no knowledge of either. In Experiment 1, Spanish and Pakistani participants completed a 5-or-not-5 task with Persian–Indian numbers. As a measure of visual similarity in Experiment 1, Garcia-Orza et al. (2012) codified a perceptual function, P_GP_ (Table 1), which was simply Spanish students' reaction time in this task. Like Cohen (2009), Garcia-Orza et al. (2012) employed N_W_ as a measure of magnitude processing. Using linear regressions, the researchers found that both P_GP_ and N_W_ separately and significantly predicted Pakistani students' reaction time; but when both factors were entered simultaneously in a multiple regression, P_GP_ was the only significant predictor. In Experiment 2, Spanish and Jordanian participants completed a 5-or-not-5 task with Arabic–Indian numbers. As a measure of visual similarity in Experiment 2, Garcia-Orza et al. (2012) codified another perceptual function, P_GA_ (Table 1), which was simply Spanish students' reaction time in this task. For Experiment 2, the researchers again employed N_W_ as a measure of magnitude processing. The researchers found that P_GA_ was the only significant predictor of Jordanian students' reaction time. Based on the results of both experiments, the researchers argued that both Persian and Arabic versions of Indian numbers do not automatically activate magnitude representations.

In a related study, Defever et al. (2012) investigated the development of magnitude representations in children aged 5 to 11 using a symbolic same–different task. As a measure of visual similarity, the researchers recruited 15 adults to provide a subjective rating for every possible combination of Arabic digit pairs using a seven point scale (Campbell & Clark, 1988). This function is abbreviated as P_SA_ (Table 1). Numerical distance between any two digits was used as a measure of magnitude representations, abbreviated here as N_D_ (Table 1). The formula was N_D_ = L–S, where L is the larger number and S is the smaller number. Using a factorial ANOVA, the researchers found a main effect of subjective visual similarity in children, such that digit pairs with high subjective visual similarity ratings were associated with longer reaction times than digit pairs with low ratings and no effect of numerical distance. These results seem to suggest that Arabic numbers do not automatically activate magnitude representations.

### 1.3 The present study

The main aim of our study was to investigate whether single-digit Arabic numbers automatically activate their corresponding magnitude representations in both adults and children by extending the work of previous studies, namely, Cohen (2009), Garcia-Orza et al. (2012) and Defever et al. (2012), particularly with respect to the experimental task, explanatory functions, participants recruited and statistical analyses.

The original 5-or-not-5 task was replaced with a symbolic same–different task: Participants would simply view pairs of single-digit Arabic numbers and judge whether they are the same or different. There were two expected advantages of this task. Firstly, the same–different task, like the 5-or-not-5 task, is appropriate to investigate whether Arabic numbers *automatically* activate magnitude representations because magnitude representations are not explicitly task-relevant. Participants may successfully complete the task using one of at least two possible strategies: by matching the numbers based on their physical properties (e.g. treating them as shapes) or on their numerical properties (which would entail magnitude processing). Secondly, this task would allow us to employ all possible combinations of Arabic numbers in the symbolic same–different task. Both Garcia-Orza et al. (2012) and Cohen (2009) employed only pairs of numbers that had the digit 5 as one of the stimuli (i.e. 5 and X) in their task. However, to fully answer the research question of whether single-digit Arabic numbers automatically activate their magnitude representation, it would be more appropriate to use *all* possible combinations of single-digit Arabic numbers.

Next, we included additional measures of visual similarity and magnitude representations. Cohen (2009) and Garcia-Orza et al. (2012) employed only one perceptual function and one numerical function in each regression analysis. In the present study, four perceptual functions and three numerical functions were employed. These functions were computed for every possible pair of digits. Following the logic of Cohen's (2009) and Garcia-Orza et al.'s (2012) experiments, we broadly reasoned that, if single-digit Arabic numbers automatically activate magnitude representations, a numerical function would best predict reaction time data. Conversely, if single-digit Arabic numbers do not automatically activate magnitude representations, then a perceptual function would best predict the data. Table 1 displays a complete list of the seven functions and their corresponding abbreviations.

The first perceptual function was P_C_, Cohen's (2009) original function (Table 1). This function was included to check for comparability with the new perceptual functions and to ensure comparability between Cohen's (2009) study and the current study.

The second perceptual function was P_CM_, a modified version of Cohen's (2009) original function (Table 1). The formula was P_CM_ = O/T, where O is the number of lines that the two digits share (i.e. overlapping lines), and T is the fewest total number of lines that are used to create the two digits (i.e. without double counting the overlapping lines) (Table 1) (Fig. 1e). There were two expected advantages of this function. Firstly, P_C_ can be computed for any digit pair consisting of two non-identical numbers; but it cannot be computed for identical numbers. For example, P_C_ for the digits 8 and 8 (i.e. P_C_ = O/D = 7/0) is a mathematical impossibility. P_CM_ would be conceptually very similar to the original P_C_ function; yet, it would allow identical number pairs to be given a value. Moreover, P_CM_ was crafted to be mathematically consistent with the third perceptual function: in both functions, visual similarity is broadly defined as the amount of overlapping area divided by the total area.

**Fig. 2:**
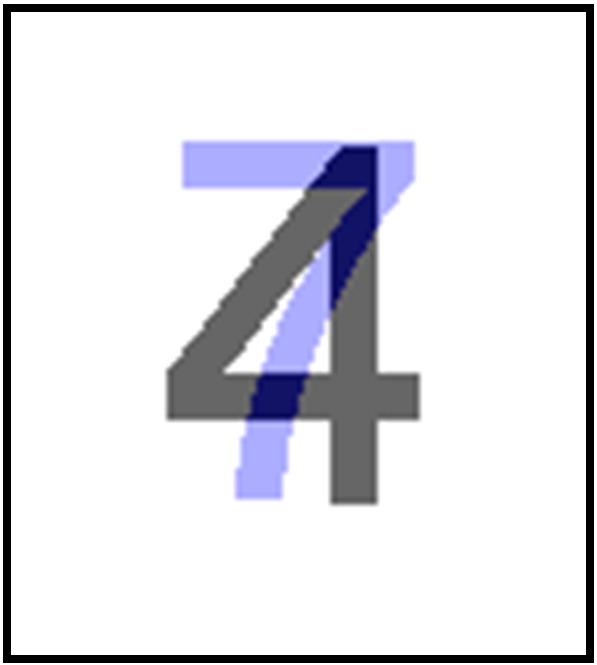
Superimposing two single-digit Arabic numbers in Arial font to calculate P_P,_ the perceptual function of pixel overlap.

The third perceptual function was P_P_, an objective measure of pixel overlap (Table 1). First, we used Adobe Photoshop CS5.1 to superimpose two Arial font single-digit numbers on each other (Fig. 2). Then, we codified the formula: P_P_ = O/T, where O is the number of overlapping pixels between the two digits, and T is the fewest total number of pixels occupied by the two digits (i.e. without double counting the overlapping pixels). This was a novel function that had not been previously employed by Cohen (2009), Garcia-Orza et al. (2012) or Defever et al. (2012). There were two expected advantages of this function. Firstly, while P_C_ and P_CM_ would be able to detect the broad shape of Arabic numbers, P_P_ was expected to be more sensitive to fine visual details. Next, P_P_ would offer consistency in font between the stimuli employed to calculate predicted reaction time (Arial font) and the actual stimuli employed in the study (Arial font). We were interested in whether such a font-consistent function would predict RT data differently from the original function P_C_ (and P_CM_), which is said to be font-independent (Cohen, 2009).

The fourth perceptual function was a subjective measure in which each participant rated the perceived visual similarity of every possible pair of single-digit Arabic numbers on a 10-point scale, from 1 (extremely different) to 10 (totally identical). This function was adapted from Campbell and Clark (1988) and Defever et al. (2012). The function was abbreviated as the perceptual function of subjective visual similarity for adults (P_SA_) and the perceptual function of subjective visual similarity for children (P_SC_), respectively (Table 1). This function was included because some aspects of visual perception might not be fully explained by the ‘objective’ functions described above. Early theories in the field of visual perception echoed this view that any visual experience might encompass an element of subjectivity. Gestalt psychologists, for example, emphasised holistic aspects of perception (Palmer, 1996) while empiricist theories interpreted visual perception in terms of sensation and association (Spelke, 1990). Moreover, Defever et al. (2012) recently found subjective visual similarity ratings to have a significant relationship with reaction time. However, the researchers used *adult* subjective visual similarity ratings to predict *children's* reaction time data. Substituting children's ratings for adults' may not have been an optimal strategy; particularly because there was no explicit evidence that adults' subjective visual similarity ratings were a valid proxy for children's ratings. Thus, in the present study, we included a subjective visual similarity function for children (P_SC_).

**Fig. 3:**
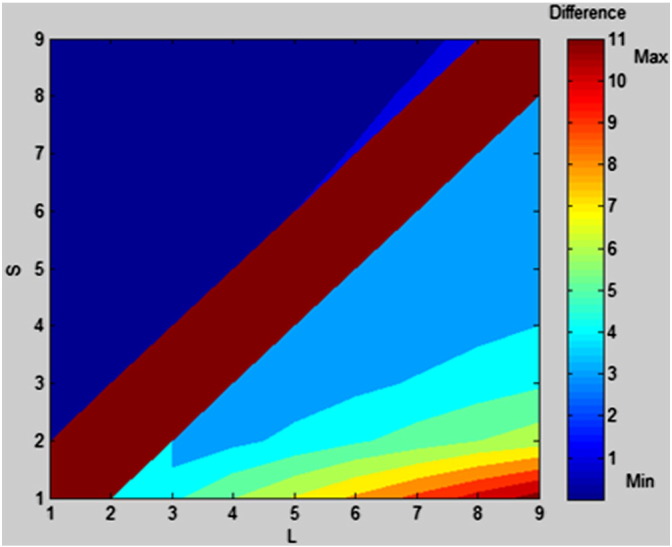
A plot of the difference between N_W_ = lg(L/L–S) and N_R_ = S/L, where L is the larger number and S is the smaller number. Both L and S are plotted for the range 1 to 9. The area of interest is L > S, i.e. all the (triangular) area to the right of the brown strip. This area varies in colour from dark blue (minimum difference, 1.e. 0) to bright red (maximum difference, i.e. 8). Dark brown represents infinity. The graph demonstrates that the two functions are highly similar (as represented by the blue areas), but not identical.

The first numerical function was N_W_, the Welford function used by Cohen (2009) and Garcia-Orza et al. (2012) (Table 1). N_W_ was included to check for comparability with the two new numerical functions. The second numerical function was based on the distance effect (N_D_) (Table 1), which is a mathematically simpler version of the Welford (1960) function. This function was employed by Defever et al. (2012). The third numerical function was based on the ratio effect, N_R_ (Table 1). The formula was N_R_ = S/L, where S is the smaller number and L is the larger number. The ratio effect is well-established as a marker of magnitude representations (Cantlon, Safford, & Brannon, 2010); yet, it was not previously employed by Cohen (2009), Garcia-Orza et al. (2012) or Defever et al. (2012). The potential advantage of including this function is that the ratio effect is sometimes argued to explain more variance in reaction time and accuracy than the numerical distance effect (Bugden & Ansari, 2011). N_R_ was computed for every possible pair of digits. To check that N_W_ and N_R_ were mathematically distinct functions, we created a 2D contour plot in MATLAB (MathWorks Inc., Natick, Massachusetts). Fig. 3 shows a plot of the difference between the two functions on a graph. The graph demonstrates that the two functions are highly similar, but not identical.

We investigated the role of visual similarity in the same–different task for both adults and children. Cohen (2009), Garcia-Orza et al. (2012) and Lyons et al. (2012) recruited adult participants only, while Defever et al. (2012) recruited children only. None of these papers investigated both adults and children in the same study. Moreover, few studies investigating this research question have recruited child participants (Defever et al., 2012; Duncan & McFarland, 1980). It was hoped that recruiting child as well as adult participants would alleviate the dearth of developmental research on this question. Moreover, this would allow us to investigate the developmental importance of physical similarity in the symbolic same–different task, which has yet not been investigated in the literature.

Finally, none of the previous studies tested for a possible interaction between functions. Both Cohen (2009) and Garcia-Orza et al. (2012) found that a perceptual and a numerical function separately and significantly predicted reaction time; yet, they did not test for an possible interaction between the functions. Thus, in the present study, we decided to test for a possible interaction between a perceptual and a numerical function, to the extent that at least one function significantly predicted reaction time.

There were five hypotheses. Previous related studies found that a perceptual, rather than a numerical, function best predicted reaction time (Cohen, 2009; Defever et al., 2012; Garcia-Orza et al., 2012; Lyons et al., 2012). Hence, they concluded that those symbolic numbers do not automatically activate magnitude representations. Based on their findings, we hypothesised that a perceptual, rather than a numerical, function would best predict reaction time in adults (Hypothesis 1). On the basis of Cohen's (2009) findings, we specifically hypothesised that P_C_ would be the best predictor of reaction time in adults (Hypothesis 2). It was possible that an interaction between a perceptual function and a numerical function would predict reaction time, but no apriori predictions were made. Since Defever et al. (2012) found a significant relationship between (subjective) visual similarity and reaction time in children, we expected the children in our sample to also show strong effects of visual similarity. Thus, we hypothesised that a perceptual, rather than a numerical, function would best predict reaction time in children (Hypothesis 3). Based on Defever et al.'s (2012) findings, we specifically hypothesised that P_SC_ would be the best predictor of reaction time in children (Hypothesis 4). It was possible that an interaction between a perceptual function and a numerical function would predict the reaction time data, but no apriori predictions were made. Finally, we hypothesised that there would be no significant differences in how adults and children use visual similarity information in the symbolic same–different task (Hypothesis 5). Although no existing study has explicitly examined the developmental importance of visual similarity in symbolic same–different task, related work suggests that 10-year-old children would, like adults, be highly adept at accessing magnitude information from symbolic numbers and would not rely on perceptual information in this task significantly more than adults would (Duncan & McFarland, 1980; Rubinsten et al., 2002; Sekuler & Mierkiewicz, 1977; Temple & Posner, 1998).

## 2 Methods

### 2.1 Participants

All participants were proficient in English and had normal or corrected-to-normal vision. The study was approved by the Psychology Research Ethics Committee of the University of Cambridge. All adult participants and the parents of child participants gave written informed consent.

#### 2.1.1 Adults

20 right-handed adults (8 males, 12 females) were recruited by posters and advertisements within the University of Cambridge to participate in this study. They ranged from 20 to 29 years old (*M* = 24.00, *SD* = 2.81). Participants were paid £7 upon completion of the experiment.

#### 2.1.2 Children

15 year 5 children (7 males, 8 females) based in Cambridgeshire, Hertfordshire and Essex were recruited by phone calls to their parents to participate in this study. They ranged from 10 to 11 years old (*M* = 10.47 years, *SD* = 0.52). Of these children, 13 were right-handed and 2 were left-handed. Upon completion of the experiment, participants received small gifts.

### 2.2 Stimuli

#### 2.2.1 Computer-based task

The display shown in each trial consisted of a pair of single-digit Arabic numbers, one on the left and one on the right of the screen. There were 480 pairs of single-digit Arabic numbers (i.e. trials) in total. In 40% of trials, the number pairs were the same (e.g. 1 and 1) (henceforth referred to as “same” trials). In the remaining 60% of trials, the number pairs were different (e.g. 9 and 2) (henceforth referred to as “different” trials). To create the “different” trials, all 36 possible pairs of single-digit Arabic numbers were used.

The proportion of ‘same’ vs. ‘different’ trials was 40:60 for several reasons. First, psychophysical research has found that, in same–different tasks with a 50% chance that the correct response is same (or different), participants are biased to responding “same” (Macmillan & Creelman, 2005; Venezia, Saberi, Chubb, & Hickok, 2012). In fact, by analysing the properties of electroencephalographic components, Soltész, Szűcs, and Szűcs (2010) established that “same” vs. “different” expectations can be approximately balanced by using a 40:60 ratio for these trials. Next, because we only analysed “different” stimuli, using 60% “different” stimuli enabled us to maximise the number of trials collected for analysis. This approach is similar to a recent study where the ratio of same to different trials was slightly more extreme than in the present study — 1:2 (i.e. approximately 33% same trials and 66% different trials) (Sasanguie et al., 2011). Furthermore, Van Opstal and Verguts (2011) have argued that the numerical distance effect in the same–different task is highly robust to differences in stimulus probability.

The order of the digits was reversed, thus giving rise to 72 different number pairs. These 72 pairs were multiplied by 4, to give a total of 288 pairs of different digits. Stimuli were presented on a 15 inch laptop computer screen. The numbers were black on a white background in Arial (size 60) font, subtending 1.89° of visual angle. The left number was presented 3.25 cm to the left of the centre of the screen, while the right number was presented 3.25 cm to the right of the centre of the screen. Adobe Photoshop CS5.1 (Adobe Systems Inc., San Jose, CA) was used to design the stimuli. Presentation (Neurobehavioral Systems Inc., San Francisco, CA) was used to programme and present the task.

#### 2.2.2 Questionnaire

Participants were given a questionnaire in which they rated the subjective visual similarity of all possible pairs of single-digit Arabic numbers. The Arabic numbers were in Arial size 20 font. Participants were also provided with an instruction sheet to help them understand the 10-point rating scale.

### 2.3 Procedures

Participants were tested individually in a quiet room at the Centre for Neuroscience in Education. There were two parts to the experiment. All participants did the computer-based task first, and then the questionnaire.

For the computer-based task, participants sat in front of a laptop and were told that they were about to play a computer game. They were instructed to decide if two numbers presented on the computer screen were the same or different and respond as quickly and as accurately as possible. This task consisted of 8 blocks.

To counterbalance the finger used to indicate same/different responses within participants, the instructions for the first four blocks differed from the instructions for the final four blocks. Participants were told to place their left index finger on the button “A” and their right index finger on the button “L”. For the first four or final four consecutive blocks of the experiment, they were to make a left button-press response (“A”) if the numbers were the same and a right button-press response (“L”) if the numbers were different. For the other four consecutive blocks, participants made a right button-press response (“L”) if the numbers were the same and a left button-press response (“A”) if the numbers were different. The order of the instructions was counterbalanced between participants. In other words, half of the participants were instructed to press “A” if the numbers were the same and “L” if they were different in the first four blocks, and “L” if the numbers were the same and “A” if they were different in the next four blocks. The other half of the participants were instructed to press “L” if the numbers were the same and “A” if they were different in the first four blocks, and “A” if the numbers were the same and “L” if they were different in the next four blocks^1^. Whenever participants received a set of new instructions (i.e. the beginning of Block 1 and Block 5), they had 5 practise trials before the actual trials began.

**Fig. 4:**
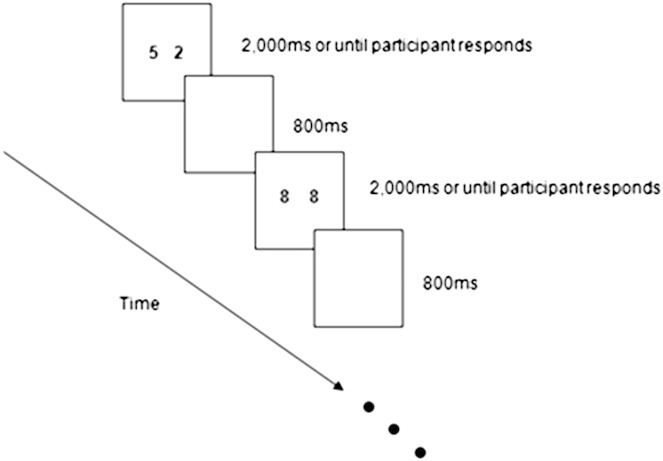
Schematic diagram of the same–different task.

Each block consisted of 60 trials. A pair of digits would be presented for 2000 ms (3000 ms for child participants) or until the participant responded with a button-press. This would be followed by a blank screen for 800 ms. The next pair of digits would be presented, and so forth until 60 trials were completed (Fig. 4). Within each block, there were 36 (60%) “different” trials and 24 (40%) “same” trials. The trials were randomly presented on the condition that there were no successive trials requiring “different” responses in which stimuli was identical. In other words, a trial with the digits “1” and “3” could not immediately follow a trial with the digits “1” and “3”. At the end of each block, the screen displayed their accuracy for that block. Participants took a short rest of 2–3 min before proceeding to the next block. The average time spent on the computer-based task, including breaks, was approximately 40 min.

Next, participants were presented with a questionnaire. They were instructed to judge the visual similarity of pairs of Arabic numbers using a 10-point scale, from 1 (extremely different) to 10 (totally identical). The terms “physical similarity” and “visual similarity” were used interchangeably. Participants were told that these judgements were subjective, so there were no fixed right or wrong answers, and that there was no time limit for this task. To provide participants with an example of a rating, they were told that, in Arial font, the visual similarity of the digits 3 and 8 was fairly high, so the numbers should be rated somewhere between 5 and 9 on the scale. Participants were given a chance to ask questions and clarify that they understood the instructions before beginning the task. All participants completed the questionnaire within 15 min. 2 A table of the participants' subjective similarity ratings for all stimulus pairs is provided in Appendix A.

### 2.4 Data analyses

#### 2.4.1 Selection criteria

Only the 288 “different” trials were analysed. Of these trials, incorrect trials (1.74% for adults and 4.91% for children) were removed. There were no outliers in the remaining trials. Additionally, there were no trials in which reaction time was below 200 ms. In deciding which participants to include in the analyses, we looked at their overall accuracy scores for the same–different task and whether they had generally understood the questionnaire on subjective visual similarity. For the same–different task, each adult participant had a good overall accuracy score of 90% and above, and each child participant had a good overall accuracy score of 80% and above. To check if participants understood the questionnaire, we inspected their ratings for identical number pairs (e.g. 3 and 3). All participants gave a rating of 10 (totally identical) for identical number pairs, indicating that they had understood the instructions. Thus, all participants were included in the analyses.

#### 2.4.2 Preliminary analyses

In the preliminary analyses, we studied the perceptual and numerical functions for all possible “different” trials, without any reaction time data. The aim of the preliminary analyses was to investigate the comparability among the perceptual functions, the comparability among the numerical functions, as well as comparability between functions used in the present study and those of previous studies.

A one-sample Kolmogorov–Smirnov test revealed that all the variables were normally distributed, except for P_C_, P_P_ and N_D_ (*p* < .05). For the purposes of the preliminary analyses only, we logarithmically transformed P_C_, P_P_ and N_D_. A one-sample Kolmogorov–Smirnov test revealed that the transformed variables were now normally distributed (*p* > .05). For the rest of the main analyses, the original non-log-transformed values of P_C_, P_P_ and N_D_ were employed. Pearson's correlations indicated a statistically significant relationship among the perceptual functions (P_C_, P_CM_, P_P_, P_SA_, P_SC_) (Table 2). This suggested that the perceptual functions were consistently tapping on visual similarity. Pearson's correlations indicated a statistically significant relationship among the numerical functions (N_W_, N_D_, N_R_) (Table 3). This suggested that the numerical functions were consistently tapping on magnitude information.

Then, we investigated the comparability of our study with that of Cohen (2009). Using number pairs of 5 and any other single-digit number apart from 5 (henceforth referred to as X′), Cohen (2009) found a significant correlation between P_C_ and N_W_ (*r* = .62). We selected number pairs of 5 and X′ in our dataset and ran a one-sample Kolmogorov–Smirnov test. For this stimulus set, the variables P_C_ and N_W_ were normally distributed (.60 < *p* < .87) and so there was no need to transform the variables. There was a significant correlation between P_C_ and N_W_ (*r* = .60, *p* = .015), demonstrating that there were no problems replicating Cohen's (2009) findings, to the extent that our stimuli and functions were identical. When all possible number pairs were used, P_C_ was not normally distributed so the log-transformed P_C_ was employed. There remained a statistically significant but weaker correlation between P_C_ and N_W_ (*r* = .24, *p* < .05). Table 4 shows a correlation matrix for all functions.

#### 2.4.3 Main analyses

There were five steps of the main analyses. First, we ran linear regressions to examine which function would best predict reaction time in adults (Hypotheses 1 & 2) and in children (Hypotheses 3 & 4). Then, we checked that the assumptions of linear regression were satisfied in the regression models for adults and for children. Next, we tested for a possible interaction between a perceptual and a numerical function in the reaction time data of adults and of children. Then, we ran a multiple regression with group × subjective visual similarity on the combined adult and child reaction time data to examine the developmental importance of visual similarity information in the symbolic same–different task (Hypothesis 5).

## 3 Results

### 3.1 Linear regressions

**Fig. 5:**
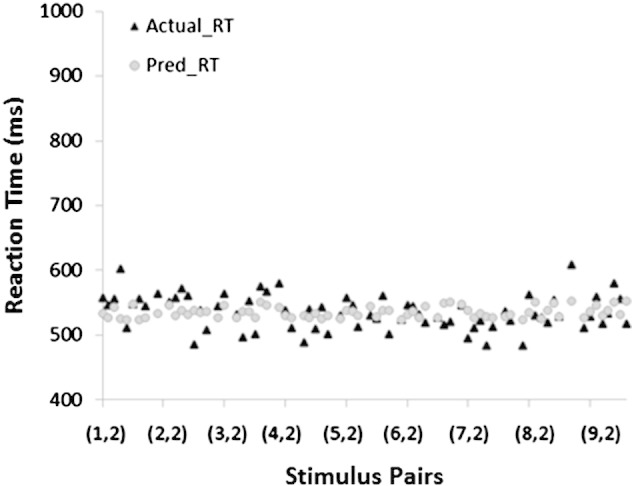
Plot of actual average RT (black triangles) and predicted RT based on P_SA_ (grey circles) for adults. On the x-axis, (x, y) indicates the pair of “different” digits that was presented. From left to right, the pairs progress from (1, 2), (1, 3), (1, 4)… (1, 9), (2, 2), etc.

**Fig. 6:**
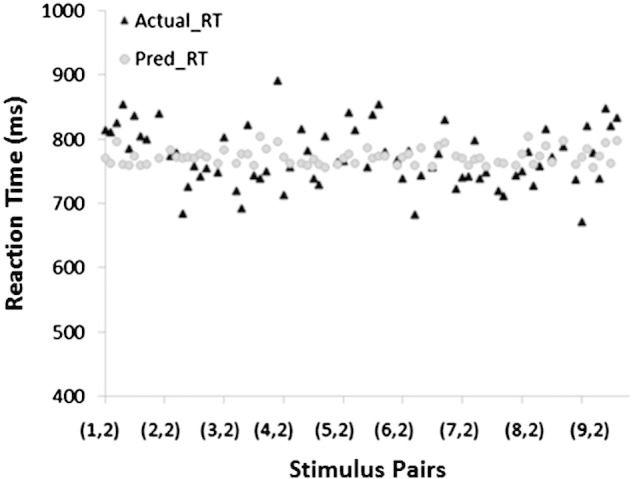
Plot of actual average RT (black triangles) and predicted RT based on P_SA_ (grey circles) for children. On the x-axis, (x, y) indicates the pair of “different” digits that was presented. From left to right, the pairs progress from (1, 2), (1, 3), (1, 4)… (1, 9), (2, 2), etc.

To find out which function(s) would predict reaction time in adults, we computed seven linear regressions. Each linear regression had one criterion variable (adults' average reaction time) and one predictor variable (P_C_, P_CM_, P_P_, P_SA_, N_W_, N_D_ or N_R_). As seen in Table 5, only P_SA_ significantly predicted the reaction time data of adults, *F*(1,70) = 8.19, *p* = .006, *R*^2^ = .11, β = .324. The other functions were not significant^3^ (.19 < *p* < .88). To find out which function(s) would predict reaction time in children, we computed seven linear regressions. Each linear regression had one criterion variable (reaction time) and one predictor variable (P_C_, P_CM_, P_P_, P_SC_, N_W_, N_D_ or N_R_). As seen in Table 6, only P_SC_ significantly predicted the reaction time data in children, *F*(1,70) = 5.57, *p* = .021, *R*^2^ = .07, β = .27. The other functions were not significant (.24 < *p* < .89). These analyses were repeated with accuracy as the criterion variable, but none of the functions significantly predicted the accuracy data in adults or in children. Figs. 5 and 6 provide a plot of the average reaction time and predicted reaction time for adults and for children based on P_SA_ and P_SC_, respectively.

### 3.2 Assumptions of linear regression

The assumptions of linearity, homoscedasticity and normality of the error distribution were satisfied for both the adult and child datasets.

White's test was used to check for the assumption of homoscedasticity. For our adult model (i.e., subjective physical similarity as the predictor and reaction time as the dependent variable), the White statistic (*R*^2^ ∗ n) was 1.00. Since *R*^2^ ∗ n < *χ*^2^ (72), the null hypothesis of homoscedasticity was not rejected. For our child model (i.e., subjective physical similarity as the predictor and reaction time as the dependent variable), the White statistic (*R*^2^ ∗ n) was 0.72. Since *R*^2^ ∗ n < *χ*^2^ (72), the null hypothesis of homoscedasticity was not rejected.

To statistically test the assumption of linearity for the adult model, we ran a regression model with two independent variables (P_SA_ and P_SA_^2^). Since P_SA_^2^'s regression coefficient had a significance value that was greater than .05 (*p* = .62), the null hypothesis of linearity was not rejected. For statistical testing of the assumption of linearity for the child model, we ran a regression model with two independent variables (P_SC_ and P_SC_^2^). Since P_SC_^2^'s regression coefficient had a significance value that was greater than .05 (*p* = .42), the null hypothesis of linearity was not rejected.

The Shapiro–Wilk test was used to check for normality of the error distribution. The assumption of normality of the error distribution was not rejected for the adult model (*p* = .39) or the child model (*p* = .50).

### 3.3 Multiple regression with subjective visual similarity × Welford function

We checked whether an interaction between a perceptual and a numerical function would predict reaction time in adults or in children, and in so doing, rigorously tested whether subjective visual similarity (P_SA_/P_SC_) was the *only* predictor of reaction time data. For the adult data, P_SA_ was selected as the perceptual function because it showed a significant relationship with reaction time. N_W_ was selected as the numerical function because, in previous studies, it was consistently found to have a significant relationship with reaction time (Cohen, 2009; Garcia-Orza et al., 2012). For the child data, P_SC_ was selected as the perceptual function because it showed a significant relationship with reaction time in children, and N_W_ was selected as the numerical function.

First, we standardised each of the predictors, P_SA_/P_SC_ and N_W_ (ZP_SA_/ZP_SC,_ ZN_W_). Next, we created the interaction term by multiplying the two standardised predictors (ZP_SA_ ∗ ZN_W_ or ZP_SC_ ∗ ZN_W_). Finally, we regressed reaction time for adults or for children on the three predictors (ZP_SA_/ZP_SC_, ZN_W_, ZP_SA_ ∗ ZN_W_ or ZP_SC_ ∗ ZN_W_) using a forced entry multiple regression. In adults, the overall model was significant, *F*(3,68) = 3.39, *p* = .023, *R*^2^ = .13, and this was probably driven by ZP_SA_ (β = .34, *t*(71) = 2.93, *p* = .005), as the interaction term (*p* = .61) and ZN_W_ (*p* = .19) were not significant. In children, the overall model was not significant (*p* = .69). Thus, there was no evidence that an interaction between a perceptual (P_SA_/P_SC_) and a numerical (N_W_) function predicted reaction time in adults or in children.

To exclude the role of any numerical comparison processes, we repeated the above analysis for N_D_. In adults, the overall model was marginally significant, *F*(3,68) = 2.70, *p* = .052, *R*^2^ = .11, and this was probably driven by ZP_SA_ (β = .32, *t*(71) = 2.79, *p* = .007), as the interaction term (*p* = .94) and ZN_D_ (*p* = .71) were not significant. In children, the overall model was significant, *F*(3,68) = 2.97, *p* = .038, *R*^2^ = .12, and this was probably driven by ZP_SC_ (β = .26, *t*(71) = 2.24, *p* = .028), as the interaction term (*p* = .14) and ZN_D_ (*p* = .21) were not significant. Thus, there was no evidence that an interaction between a perceptual (P_SA_/P_SC_) and a numerical (N_D_) function predicted reaction time in adults or in children.

We also repeated the analysis for N_R_. In adults, the overall model was significant, *F*(3,68) = 3.43, *p* = .022, *R*^2^ = .13, and this was probably driven by ZP_SA_ (β = .35, *t*(71) = 3.01, *p* = .004), as the interaction term (*p* = .72) and ZN_D_ (*p* = .17) were not significant. In children, the overall model was not significant (*p* = .13). Thus, there was no evidence that an interaction between a perceptual (P_SA_/P_SC_) and a numerical (N_R_) function predicted reaction time in adults or in children.

In other words, the final model that best described the adult and child data was a linear regression with reaction time as the criterion variable and adult/child subjective ratings of visual similarity respectively as the predictor.

### 3.4 Multiple regression with group × subjective visual similarity

To test Hypothesis 5, we investigated the developmental importance of visual similarity by running a forced entry multiple regression on the combined adult and child reaction time data with three standardised predictors: group (children: 0, adults: 1), subjective visual similarity (P_SA_/P_SC_), and an interaction between group and subjective visual similarity. The logic of this analysis is that the interaction term tests whether there is a difference in the beta value of the subjective visual similarity variable for adults and for children. In other words, a statistically significant interaction term would strongly suggest that there are significant differences between how 10-year-old children and adults use visual information in the symbolic same–different task. The overall model was significant, *F*(3, 140) = 514.00, *p* < .001, *R*^2^ = .92 and this was driven by subjective visual similarity (β = .11, *t*(143) = 2.90, *p* = .004) and group (β = − .93, *t*(143) = − 14.67, *p* < .001). The interaction (*p* = 42) term was not significant.

## 4 Discussion

### 4.1 Single-digit Arabic numbers do not automatically activate magnitude representations

In the present study, we investigated whether single-digit Arabic numbers automatically activate magnitude representations in adults and in children using the symbolic same–different task. Four perceptual functions (P_C_, P_CM_, P_P_, P_SA_ and P_SC_) and three numerical functions (N_W_, N_D_, N_R_) were employed. We hypothesised that a perceptual function would best predict reaction time in adults (Hypothesis 1) and in children (Hypothesis 3). Specifically, we expected P_C_ to be the best predictor of reaction time in adults (Hypothesis 2), and P_SC_ to be the best predictor of reaction time in children (Hypothesis 4). We found that subjective visual similarity (P_SA_ and P_SC_) was the best and only significant predictor of reaction time in adults (*p* = .006, *R*^2^ = .11, β = .324) and in children (*p* = .021, *R*^2^ = .07, β = .27), respectively. Therefore, Hypotheses 1, 3 and 4 were supported and Hypothesis 2 was rejected. These results strongly suggest that, in this task, single-digit Arabic numbers do not automatically activate magnitude representations in adults or in children. This conclusion concurs with those of recent studies, namely, Cohen (2009), Garcia-Orza et al. (2012), Lyons et al. (2012) and Defever et al. (2012). Additionally, our results concur with Defever et al. (2012) on the likely importance of subjective visual similarity in the symbolic same–different task.

However, there are two ways in which our results differ from, and, in fact, extend Defever et al.'s (2012) findings. Firstly, Defever et al.'s (2012) work demonstrated the importance of subjective visual similarity in children's reaction time data in the symbolic same–different task. Our results extend theirs by demonstrating the importance of subjective visual similarity to reaction time in the symbolic same–different task in both children and adults. Next, Defever et al. (2012) used *adult* subjective ratings of visual similarity to predict *children's* reaction time data. This may not have been be an optimal strategy, particularly because the researchers did not provide any evidence that adults' subjective ratings were a valid proxy for children's ratings. Our results explicitly demonstrate that 10-year-old *children's* subjective ratings of visual similarity significantly predict their own reaction time data, and this confirms Defever et al.'s (2012) conclusions on the role of subjective visual similarity in the symbolic same–different task in children.

### 4.2 Similarities between adults and children

There were striking similarities between the adult and child data. As already discussed, subjective visual similarity (P_SA_ and P_SC_) was the best and only significant predictor of reaction time in the symbolic same–different task for both adults and children. Next, in support of Hypothesis 5, we found no significant differences between how 10-year-old children and adults use visual information in the symbolic same–different task, as evidenced by the statistically insignificant interaction term in the subjective visual similarity × group multiple regression. Furthermore, the subjective visual similarity ratings for adults and for children were highly correlated (*r* = .91, *p* < .001) (Table 1), suggesting that 10-year-old children are already able to give a subjective rating for visual similarity that is highly similar to that of mature adults. These findings provide support for Defever et al.'s (2012) decision to substitute the subjective visual similarity ratings of children for those of adults. Overall, children responded more slowly than did adults in the symbolic same–different task, as seen in Figs. 5 and 6, and the statistically significant group term (*p* < .001) in the group × subjective visual similarity multiple regression.

### 4.3 Why did P_C_ fail to significantly predict reaction time?

In Cohen's (2009) study, P_C_ was the best predictor of reaction time. However, in the present study, it was not even a significant predictor of reaction time in adults or in children. Earlier, we suggested that a possible weakness of the function P_C_ was its potential lack of ecological validity. Specifically, there was an inconsistency of font between the function P_C_ (figure 8 structure) and the actual font in the experiment (Arial font). Why, then, did Cohen (2009) find a significant relationship between P_C_ and reaction time? It is possible that the limited set of stimuli in Cohen's (2009) study (i.e. 5 and X) might have made it easier to obtain a significant relationship. In the present study, all possible combinations of single-digit Arabic number pairs were employed, which provided a test of whether the alleged relationship between P_C_ and reaction time in the symbolic same–different task would still hold. We did not find evidence for such a relationship in adults or in children.

### 4.4 The relationship between visual similarity and magnitude representations

Cohen (2009) previously proposed that visual similarity and magnitude representations share a close relationship, based on his finding that the correlation between P_C_ and N_W_ for single-digit number pairs of 5 and X′ (i.e. any single-digit number apart from 5) was *r* = .62. We replicated this finding in the preliminary analyses: the correlation between P_C_ and N_W_ for single-digit number pairs of 5 and X′ was *r* = .60. Thus, we could replicate Cohen's (2009) findings when using identical stimuli. However, the correlation between P_C_ and N_W_ was much lower than expected when the full set of stimuli was employed (*r* = .24, *p* = .039).

Our study distinguished between “objective” measures of visual similarity (P_C_, P_CM_, P_P_) and “subjective” measures of visual similarity (P_SA_ and P_SC_). In our study, the “objective” measures of visual similarity (P_CM_, P_P_) had a significant relationship with magnitude information (as operationalised by N_W_). In contrast, the subjective measures of visual similarity (P_SA_, P_SC_) significantly correlated with the “objective” measures, but not with magnitude information (N_W_) (Table 1). This suggests that the subjective ratings of visual similarity by adults and children, relative to “objective” measures, provided a different measure of visual similarity and was uncontaminated by magnitude information.

### 4.5 What is driving subjective visual similarity?

In the present study, the subjective visual similarity function was the best and only significant predictor of reaction time. This raises a new question for future research to address: what is driving the subjective visual similarity function?

One possibility is that curves and straight lines that form acute angles might drive subjective visual similarity. There exists evidence that our visual system distinguishes between curves and angles, and that this information can transfer across other sensory systems. For example, Ramachandran and Hubbard (2001) reported that young adults in America and in India tend to associate angular edges with sharp sounds like “kiki” and rounded edges with round sounds like “bouba”. Similarly, Parise and Spence (2009) found that people tend to associate angular shapes with high-pitched sounds and rounded shapes with lower-pitched sounds. Certainly, such an explanation remains speculative for now, and future research is needed to clarify the source of subjective visual similarity.

### 4.6 The role of perceptual properties in tasks of numerical cognition

Our results call for a deeper consideration of the role of perceptual properties in putative tasks of numerical cognition. In the present study, we found evidence for the influence of subjective visual similarity on the symbolic same–different task. Recent studies have demonstrated growing awareness of the relationship between perceptual properties and non-symbolic number tasks (e.g. Burr, Anobile, & Turi, 2011; Burr & Ross, 2008; Gebuis & Reynvoet, 2012a; Gebuis & Reynvoet, 2012b; Paffen, Plukaard, & Kanai, 2011; Ross & Burr, 2010; Soltész et al., 2010; Szűcs, Nobes, Devine, Gabriel, & Gebuis, 2013; Szűcs & Soltesz, 2008). Our results contribute to this literature by demonstrating that symbolic, and not just non-symbolic, number judgements are influenced by perceptual cues. Additionally, our results demonstrate that the role of visual perception in symbolic number judgements is influential in both adults and children.

### 4.7 Conclusion

The present study found evidence to support the argument that single-digit Arabic numbers do not automatically activate magnitude representations in adults or in children. Our results demonstrated the likely role of subjective visual similarity for both adults and children in the symbolic same–different task, and more broadly, in putative tasks of numerical cognition. As the first study to date to explicitly study (by directly comparing child and adult groups) the developmental importance of perceptual factors in the symbolic same–different task, we found no significant differences between adults and 10-year-old children in their reliance on perceptual information in this task. Finally, we suggested that subjective ratings of visual similarity may be a more valid measure of perceived visual similarity than the “objective” measures based on quantifying physical properties of stimuli.

## Figures and Tables

**Table:** 

Stimulus pair		Average subjective similarity rating by children	Average subjective similarity rating by adults	Average subjective similarity rating by new independent sample of adults
1	2	3.4	4.25	2
1	3	2.33	2.95	1.36
1	4	6.73	6.15	4.79
1	5	2.07	2.6	1.5
1	6	1.93	2.15	1.29
1	7	3.8	7.3	5.43
1	8	1.87	2.25	1.79
1	9	2.2	2.8	2.14
2	1	3.4	4.25	2
2	3	5.07	6.9	6.5
2	4	3.6	3.65	2.36
2	5	3.33	5.25	4
2	6	3.53	4	3.5
2	7	3.47	5.4	3.93
2	8	4.27	4.45	4.07
2	9	3.6	4.75	4.29
3	1	2.33	2.95	1.36
3	2	5.07	6.9	6.5
3	4	2.4	2.95	1.57
3	5	4.2	4.85	4.29
3	6	4.2	4.95	4.64
3	7	2	2.8	1.93
3	8	7.8	8.05	7.57
3	9	5.33	6.9	5.86
4	1	6.73	6.15	4.79
4	2	3.6	3.65	2.36
4	3	2.4	2.95	1.57
4	5	2.33	3.45	2
4	6	1.93	3.05	1.79
4	7	3.13	4.2	3.5
4	8	2.07	2.4	1.93
4	9	1.47	3.55	2.36
5	1	2.07	2.6	1.5
5	2	3.33	5.25	4
5	3	4.2	4.85	4.29
5	4	2.33	3.45	2
5	6	5.47	6.65	5.93
5	7	3.33	3.35	2.21
5	8	3.8	5.35	4.57
5	9	3.8	5.3	4.07
6	1	1.93	2.15	1.29
6	2	3.53	4	3.5
6	3	4.2	4.95	4.64
6	4	1.93	3.05	1.79
6	5	5.47	6.65	5.93
6	7	1.8	2.95	1.64
6	8	5.87	7.65	7.29
6	9	6.47	7.8	8
7	1	3.8	7.3	5.43
7	2	3.47	5.4	3.93
7	3	2	2.8	1.93
7	4	3.13	4.2	3.5
7	5	3.33	3.35	2.21
7	6	1.8	2.95	1.64
7	8	2.53	3.15	1.86
7	9	2.33	3.85	3.5
8	1	1.87	2.25	1.79
8	2	4.27	4.45	4.07
8	3	7.8	8.05	7.57
8	4	2.07	2.4	1.93
8	5	3.8	5.35	4.57
8	6	5.87	7.65	7.29
8	7	2.53	3.15	1.86
8	9	6.87	8.2	7.79
9	1	2.2	2.8	2.14
9	2	3.6	4.75	4.29
9	3	5.33	6.9	5.86
9	4	1.47	3.55	2.36
9	5	3.8	5.3	4.07
9	6	6.47	7.8	8
9	7	2.33	3.85	3.5
9	8	6.87	8.2	7.79

**Table 1:** A list of functions and their corresponding abbreviations in this paper.

Abbreviation	Name of function	Previously used by	Formula (for details, refer to individual paragraphs)
P_C_	Perceptual function by Cohen (2009)	Cohen (2009)	P_C_ = O/D
P_GP_	Perceptual function by Garcia-Orza et al. (2012) for Persian–Indian numbers	Garcia-Orza et al. (2012)	Spanish students' reaction time for Persian–Indian numbers
P_GA_	Perceptual function by Garcia-Orza et al. (2012) for Arabic–Indian numbers	Garcia-Orza et al. (2012)	Spanish students' reaction time for Arabic–Indian numbers
P_CM_	Perceptual function by Cohen (2009) after modification	Novel	P_CM_ = O/T
P_P_	Perceptual function based on pixel overlap	Novel	P_P_ = O/T
P_SA_	Perceptual function based on subjective visual similarity ratings of adults	Campbell and Clark (1988) (adapted)	Adult participants' ratings of subjective visual similarity
P_SC_	Perceptual function based on subjective visual similarity ratings of children	Novel	Child participants' ratings of subjective visual similarity
N_W_	Numerical function based on the Welford function	Cohen (2009) and Garcia-Orza et al. (2012)	RT = a + k ∗ lg(L/L–S)
N_D_	Numerical function based on the distance effect	Defever et al. (2012)	N_D_ = L–S
N_R_	Numerical function based on the ratio effect	Novel	N_R_ = S/L

Note: O refers to the amount of overlap between any two given numbers; D refers to the difference between these two numbers; T refers to total area covered by the numbers; L refers to the larger number; S refers to the smaller number; RT refers to reaction time; a and k are constants.

**Table 2:** Pearson's correlations for perceptual functions.

	P_C_	P_CM_	P_P_	P_SA_	P_SC_
P_C_	*r* = 1				
P_CM_	*r* = .969⁎⁎⁎ *p* < .001	*r* = 1			
P_P_	*r* = .683⁎⁎⁎ *p* < .001	*r* = .713⁎⁎⁎ *p* < .001	*r* = 1		
P_SA_	*r* = .590⁎⁎⁎ *p* < .001	*r* = .622⁎⁎⁎ *p* < .001	*r* = .688⁎⁎⁎ *p* < .001	*r* = 1	
P_SC_	*r* = .485⁎⁎⁎ *p* < .001	*r* = .513⁎⁎⁎ *p* < .001	*r* = .676⁎⁎⁎ *p* < .001	*r* = .91⁎⁎⁎ *p* < .001	*r* = 1

Note: To reduce visual clutter, the correlations for any two measures is displayed only once in the matrix.

⁎⁎⁎ Correlation is significant at the 0.001 level (2-tailed).

**Table 3:** Pearson's correlations for numerical functions.

	N_W_	N_D_	N_R_
N_W_	*r* = 1		
N_D_	*r* = − .837⁎⁎⁎ *p* < .001	*r* = 1	
N_R_	*r* = .965⁎⁎⁎ *p* < .001	*r* = − .843⁎⁎⁎ *p* < .001	*r* = 1

Note: To reduce visual clutter, the correlations for any two measures is displayed only once in the matrix.

⁎⁎⁎ Correlation is significant at the 0.001 level (2-tailed).

**Table 4:**
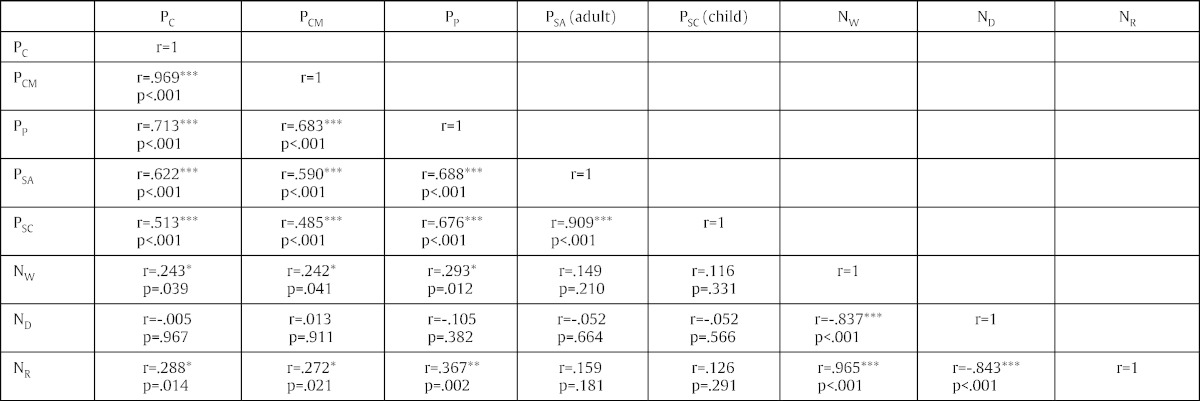
Pearson's correlations for all measures.

**Table 5:** Results of linear regressions for adult data.

	Β	*p*
P_C_	.018	.883
P_CM_	.076	.528
P_P_	.145	.191
P_SA_	.324	.006⁎⁎
N_W_	− .098	.411
N_D_	− .063	.599
N_R_	− .104	.383

⁎⁎ Correlation is significant at the 0.01 level (2-tailed).

**Table 6:** Results of linear regressions for child data.

	Β	*p*
P_C_	.063	.597
P_CM_	.016	.894
P_P_	.030	.803
P_SC_	.271	.021⁎
N_W_	.039	.743
N_D_	− .142	.236
N_R_	.041	.734

⁎ Correlation is significant at the 0.05 level (2-tailed).

## Footnotes

1 As requested by a Reviewer, we ran several t-tests for dependent samples and found no significant effect of the button switch on reaction time for adults (*p* = .97) or for children (*p* = .75).

2 In order to confirm the general nature of participant ratings, we recruited an additional group of 15 young adults who had not taken part in this study. They were asked to complete the questionnaire only; i.e. they rated the visual similarity of stimuli. The subjective similarity ratings for the original sample of adults and the new independent sample were highly correlated (*r* = .97, *p* < .001). A table of the participants' subjective similarity ratings for all stimulus pairs is provided in Appendix A.

3 As requested by a Reviewer, we used Cohen's (2010) modified formula as an additional perceptual function with which to predict reaction time in adults and in children. For the adult data, we computed a linear regression with one criterion variable (adults' average reaction time) and one predictor variable (Cohen's modified formula). For the child data, we computed a linear regression with one criterion variable (children's average reaction time) and one predictor variable (Cohen's modified formula). Cohen's (2010) modified formula did not significantly predict the reaction time data of adults (*p* = .77) or children (*p* = .62).
